# Trimester-Specific Reference Ranges for Saturated, Monounsaturated and Polyunsaturated Fatty Acids in Serum of Pregnant Women: A Cohort Study from the ECLIPSES Group

**DOI:** 10.3390/nu13114037

**Published:** 2021-11-12

**Authors:** Carla Martín-Grau, Ramón Deulofeu, Nuria Serrat Orus, Victoria Arija, on behalf of the ECLIPSES Study Group

**Affiliations:** 1 Clinical Chemistry and Laboratory Medicine, Institut Català de la Salut, Generalitat de Catalunya, University Hospital Joan XXIII, 43005 Tarragona, Spain; carla.mg324@gmail.com (C.M.-G.); nserrat.tarte.ics@gencat.cat (N.S.O.); 2 Nutrition and Mental Health Research Group (NUTRISAM), Faculty of Medicine and Health Science, Universitat Rovira i Virgili, 43201 Reus, Spain; 3 Department of Biochemistry and Molecular Genetics, Biomedical Diagnostic Center, Hospital Clínic Universitari, CiberEHM, 08036 Barcelona, Spain; deulofeu@clinic.cat; 4 Pere Virgili Institute for Health Research (IISPV), Universitat Rovira i Virgili, 43003 Tarragona, Spain; 5 Tarragona-Reus Research Support Unit, Jordi Gol Primary Care Research Institute, 43003 Tarragona, Spain

**Keywords:** individual c14-c24, fatty acid status, maternal nutrition, pregnancy, reference intervals

## Abstract

In the course of pregnancy, increasing importance is being placed on maintaining optimal fatty acid (FA) levels and particularly n-3 PUFAs to ensure correct fetal development. However, reference ranges for FA have been reported in only a few studies. Our objective is to provide quantitative reference intervals for SFAs, MUFAs, and PUFAs (n-6 and n-3) in a large population of healthy pregnant women from a developed country. A prospective study of pregnant women (*n* = 479) was conducted from the first trimester (T1) to the third trimester (T3). A total of 11 fatty acids were analyzed in serum by gas chromatography mass spectrometry and were expressed as absolute (µmol/L) and relative (percentage of total FA) concentration units. Serum concentrations of SFAs, MUFAs, n-6 PUFAs, n-3 PUFAs, various FA ratios, and the EFA index were determined. The reference intervals (2.5/97.5 percentiles) in absolute values from T1 ranged from 1884.32 to 8802.81 µmol/L for SFAs, from 959.91 to 2979.46 µmol/L for MUFAs, from 2325.77 to 7735.74 µmol/L for n-6 PUFAs, and from 129.01 to 495.58 µmol/L for n-3 PUFAs. These intervals mainly include the values of other studies from European populations. However, reference ranges vary according to some maternal factors. The FA levels proposed, obtained from a large sample of pregnant women, will be a useful tool for assessing the degree of adequacy of FAs in pregnant women and will help to carry out dietary interventions based on certain maternal factors.

## 1. Introduction

Maternal diet during the periconceptional, pregnancy and lactation period is important for both mother and child [1,2], and specifically, the fatty acid (FA) levels play a crucial role during pregnancy [3,4,5]. Eicosapentaenoic acid (EPA) and docosahexaenoic acid (DHA) are specially critical regarding central nervous system, retinal photoreceptors, and immune systems development [6]. Correct concentrations (or status) both in the first trimester (T1) and the third trimester (T3) of pregnancy are of great importance considering their role during the onset of neurogenesis [7,8], and the fetal brain development [8]. Consequently, maternal serum FA levels change in the course of pregnancy, depending on fetal requirements. In this context, it is necessary to monitor FA concentration values throughout pregnancy so that deficiency can be detected. Based on this, reference ranges are the most useful tool to evaluate the adequacy of nutritional status [9], and even in future studies these values can be used to identify women who are at risk of an adverse health outcome.

In the little literature that does exist, reference ranges for individualized FA in serum are normally reported in non-pregnant adults [10,11,12,13]. Apparently, North European populations have a higher content of total saturated fatty acids (SFAs) and a lower content of monounsaturated fatty acids (MUFAs) circulating in serum [13,14,15] than North American populations [11,12]. Furthermore, in populations of healthy pregnant women, serum reference values have only been described in a Brazilian cohort of 225 pregnant women [16] and in a Norwegian cohort of 247 pregnant women [17]. All these populations show different dietary patterns from the one in our study. In the Mediterranean population, the composition of the diet is well-known for its low content of SFAs, and high content of MUFAs and n-3 PUFAs, mainly derived from olive oil and fish consumption [18,19]. Consequently, reference values of circulating FA should be measured in different populations based on their lifestyle.

Additionally, FAs can be measured in serum, erythrocyte, or adipose tissue. It is well-recognized that FA levels in serum reflect short-term intake [20], and are more representative of the current dietary habits of subjects [21]. However, other authors have analyzed FAs in the erythrocyte membrane for assessing FA status as it reflects the last 30–60 days of intake [7,17] so comparisons among studies are difficult. In previous studies, the analysis of an individual FA may be found expressed either quantitatively or qualitatively as a relative percentage of total FAs. Without standardizing the criteria for providing an appropriate reference range, there is a great risk of misinterpretation. In this regard, the problem of presenting findings such as relative values is that they are difficult to compare since percent composition values depend on the set of individual FAs investigated [10,11]. By contrast, absolute values are not dependent upon the relative abundance of other FAs and the measurement of an individual FA reduces analysis costs thus could be included in routine clinical practice of laboratories and not be limited to research studies. A quantitative approach may potentially be more appropriate for analyzing whether FAs are associated with the risk of an adverse health outcome.

To support the measurement of individual FAs, the use of n-6/n-3, arachidonic acid (AA)/EPA, AA/(EPA + DHA) and linoleic acid (LA)/Dihomo-γ-linolenic acid (DHGLA) ratios and the essential fatty acid (EFA) index are also helpful in assessing the degree of adequacy of FAs. The increase in the AA/EPA ratio has been positively correlated with pro-inflammatory eicosanoids and associated with metabolic diseases [22,23]. An imbalance between AA and EPA + DHA was found to be associated to preterm delivery [22,24]. High LA/DHGLA ratios indicate an enzymatic deficit of delta-6-desaturase activity. Delta-6-desaturase is predominantly involved in the PUFA biosynthesis pathway and converts LA and alpha-lipoic acid (ALA) into their metabolites DHGLA, AA, EPA and DHA [3,7], although it can also catalyze palmitic acid and stearic acid thus affecting MUFA levels [25]. The n-6/n-3 ratio reflects the predominant PUFA family in serum and the formation of inflammatory mediators, which play important roles in pregnancy pathologies [26,27]. According to the available data, no study has reported reference intervals for serum n-6/n-3, AA/EPA, AA/(EPA + DHA) and LA/DHGLA ratios, or the EFA index in pregnant women. While providing these reference ranges, future studies will allow correlating newborn adverse outcomes with an individual FA abnormality or any ratio imbalance.

To our knowledge, no study has evaluated maternal FAs status and ratios in European Mediterranean countries, and including a large majority of SFAs, MUFAs and PUFAs. Considering the lack of reference intervals for pregnant women in the literature and the absence of interpretative criteria, the aim of this study was to provide the reference intervals for serum SFA, MUFA and PUFA (n-6 and n-3) in a large population of healthy pregnant women from a Mediterranean country in T1 and T3 of gestation, expressed both as concentrations and as percentages of the total concentration of FA in serum samples.

## 2. Materials and Methods

### 2.1. Population and Study Design

A prospective study of pregnant women was conducted from T1 to T3 (12 ± 0.5 and 36 ± 0.4 gestational week, respectively). Participants were healthy pregnant women from the ECLIPSES study [28,29] which is a randomized, triple-blind clinical trial of different doses of iron supplementation (20 mg/day, 40 mg/day 80 mg/day), registered in ClinicalTrials.gov (accessed on 9 November 2021) identification number NCT03196882, and in the European Union (EU) Clinical Trial Register, EUCTR-2012-005480-28. The present study was approved by the Clinical Research Ethics Committee of the Jordi Gol Institute for Primary Care Research (IDIAP) and the Pere Virgili Institute for Health Research (IISPV). Informed consent was obtained from all individuals included in this study.

**Figure 1:**
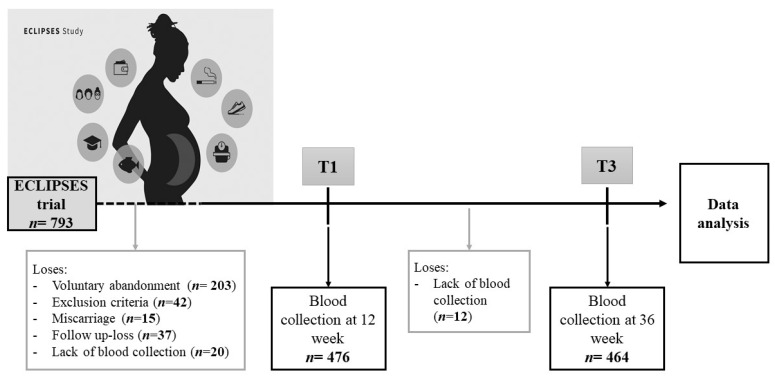
Flow chart of the study population.

The flow of participants and data is outlined in Figure 1. The inclusion criteria were as follows: healthy adult women older than 18 years at ≤12 weeks of gestation, who were able to understand the local languages (Spanish or Catalan) and the characteristics of the study. Some of the medium risk criteria and all the high risk criteria described in the Catalan pregnancy monitoring protocol [30] were used as exclusion criteria such as multiple pregnancies, underweight, morbid obesity, previous severe disease (immunosuppression), or any chronic disease that could affect nutritional development (cancer, pre-gestational diabetes mellitus, malabsorption, chronic hepatitis and liver cirrhosis).

The medical, socioeconomic, educational level and lifestyle information is shown in Table 1. The socioeconomic level was calculated by using the Catalan classification of occupations (CCO-2011) [31] such as student, employed and unemployed. The education level was classified as primary (low), high school (medium) and university studies or more (high). Physical activity was assessed by using the International Physical Activity Questionnaire [32] and was summed to obtain the total physical activity in metabolic equivalents (METs)/week. Based on total physical activity, participants were classified into 3 levels: low (<600 METs/week), moderate (≥600–2999 METs/week) and high (≥3000 METs/week). BMI was calculated and classified by following WHO criteria [33]: normal weight (BMI = 18.5–24.9 kg/m^2^), overweight (BMI = 25.0–29.9 kg/m^2^) and obesity (BMI ≥ 30 kg/m^2^) at T1. The Spanish diet quality index (SQDI) [34] was estimated from nine food groups (protein foods, dairy foods, cereals, fruits, vegetables, oil, legumes, tubers, sweets). A score was obtained ranging from 0 points (low quality diet) to 18 points (high quality diet). Women were then classified into two categories: low-medium diet quality (score from 0 to 10) and high diet quality (score from 11 to 18). Further information can be found in our previous paper [29].

### 2.2. Sample Preparation and GC-MS Conditions

#### 2.2.1. Extraction, Transfer and Storage of Biological Samples

Serum samples were collected both T1 and T3 after fasting into 7.5 mL tubes without anticoagulant and were left without mixing for 30 min at room temperature to enable coagulation. The serum was separated by centrifugation and stored into aliquots of 500 μL at −80 °C inside the BioBank. Samples were thawed at the end of the study and processed simultaneously to minimize inter-batch variation [28].

#### 2.2.2. Sample Preparation and GC-MS Conditions

The method starts with a 50 µL serum sample mixed with IS and the derivatization reagent (chloroform and methanolic hydrochloric acid). Then the sample is heated and mixed at 80 °C for 2 h. In this step, the lipidic fraction in blood or serum consisting of free FAs, sterol esters, glycerol esters (mainly triglycerides), and phospholipids were hydrolyzed and methylated into fatty acid methyl esters (FAMEs). Three sequential heating and mixing steps are done every 10 min during a 30-min period. After this, a 100 µL of iso-octane was added and the fatty acid methyl esters were extracted by a liquid-liquid extraction using hexane, and analyzed by gas chromatography mass spectrometry (GC-MS) combination using the 7890A GC coupled to triple quadrupole MS QQQ 7000 Series^®^ (Agilent Technologies Inc., Santa Clara, CA, USA). Chromatographic analysis was based on David et al. [35] to determine the 36 FAMEs included in the Food Industry FAME Mix (Restek Corporation). Briefly, the FAMEs were separated into a high-polarity column (100 m × 250 μm × 0.25 μm) (HP-88 column, Agilent Technologies) using a temperature program ranging between 140 and 240 °C at a 10°/min pace using helium as the carrier gas at 1 mL/min. Ionization was carried out by electronic impact (70 eV), and the mass analyzer was operated in Selected Ion Monitoring (SIM) mode. The CG-MS system was controlled by the Agilent MassHunter^®^ Workstation.

### 2.3. Data Analysis

A total of 36 FAs were analyzed although FA values below the limit of detection were not shown. In addition, a selection of FAs is presented such as the sum of the total saturated (Σtotal SFA = C:12 + C:14 + C:16 + C18:0), total monounsaturated (Σtotal MUFA = C16:1n-7 + C18:1n-9), total n-6 polyunsaturated (Σtotal n-6 PUFA = C18:2n-6 + C20:3n-6 + C20:4n-6) and total n-3 polyunsaturated (Σtotal n-3 PUFA = C20:5n-3 + C22:6n-3) fatty acids; furthermore, some index were also calculated such as the n-6 to n-3 fatty acids ratio (Σtotal n-6 PUFA/Σtotal n-3 PUFA) and the essential fatty acids (EFA) index (sum of the (essential) n-3 and n-6 PUFAs/sum of the (non-essential) n-7 and n-9 FAs), which reflects the overall EFA status.

FAs were expressed as absolute (µmol/L) or relative (percentage of total FA) concentration units. The method for calculating the percentage of total fatty acids is based on total FA concentration. To assist in the evaluation of overall nutritional status, reference intervals were determined following the Clinical and Laboratory Standards Institute (CLSI) C28-A3 guidelines [9] and represented the central 95% of the tested population (being the 2.5% and 97.5% confidence intervals the lower and upper limits, respectively). The FA values were analyzed using Agilent MassHunter^®^ Quantitative Analysis B.06 (Agilent Technologies Inc., Santa Clara, CA, USA). The results were expressed as mean ± standard deviation (SD) for normally distributed data. In this study, z-score analysis was used to detect outlier values in the population data [36]. An absolute z-score value above or below ±3.29 is considered to be an outlier when the sample size is >100 [37]. Correlations between the absolute and relative concentrations of FA in serum were computed using Pearson’s correlation coefficient test. All multiple linear regression models were performed using the ENTER method for total FAs, total SFA, total MUFA, total n-6 PUFA, total n-3 PUFA, LA, DHA and AA to evaluate the relation between maternal factors and FA levels. The models were adjusted for maternal factors, such as maternal age, occupation (student, employed, unemployed), educational level (low, medium, high), ethnicity (Spanish, non-Spanish women), parity (no, yes), initial BMI (Kg/m^2^), gestational weight gain (Kg/m^2^), maternal smoking status (no, yes), maternal alcohol consumption (no, yes), physical activity in METs/week (score), and SQDI (score). Data was processed using the statistical software package SPSS version 25.0 for Windows and Microsoft Excel 2016 (Microsoft Corporation, Redmond, WA, USA). A *p*-value < 0.05 was considered to be statistically significant.

## 3. Results

### 3.1. Participants’ Characteristics

The general characteristics of the pregnant women participating in the ECLIPSES study are given in Table 1. A total of 455/476 women answered the questions about sociodemographic and lifestyle characteristics. Most women had a medium educational level (38.3%) and were employed (87.1%). The maternal age was 30.6 ± 5.01 years old. The participants reported that 15.3% smoked and 14% drank at the beginning of pregnancy. Regarding physical activity, 56.4% of the women had a low level.

### 3.2. Fatty Acid Status in Serum of Pregnant Women

This study determined saturated, mono- and polyunsaturated FAs in 476 maternal serum samples at T1 and T3. A total of 36 FAs were analyzed although only 11 FAs were detected in serum of pregnant women and were summarized in Table 2 and Table 3. The FA composition in maternal serum is represented as absolute (Table 2) and relative (Table 3) FA concentrations (mean ± SD) in both T1 and T3. The absolute total amount of FAs in maternal serum increased during pregnancy (T1, 10073.15 µmol/L and T3, 20,480.82 µmol/L, *p* < 0.01) and the highest individual FAs were C16:0, C18:1n-9 and C18:2n-6 at both T1 and T3.

To compare the two reporting schemes, Pearson’s correlations coefficients were calculated between FA values expressed in absolute and relative terms for the whole sample, which ranged from 464–476 serum samples, according to data available both in T1 and T3 of pregnancy (Supplementary Table S1). In T1, significant and positive correlations (*p* < 0.01) were observed for C12:0, C14:0, C16:0, C18:2n-6, C20:3n-6, C20:4n-6, C20:5n-3, C22:6n-6, Σtotal SFA, Σtotal n-6 PUFA and Σtotal n-3 PUFA. However, the Pearson’s correlation coefficient was moderate (r < 0.5) for the most of FAs in T1 with the exception of C16:0 (r = 0.676), C20:5n-3 (r = 0.859) and Σtotal SFA (r = 0.541) for which a strong correlation was observed. In contrast, non-significant correlations were observed for C18:0, C16:1n-7, C18:1n-9 and Σtotal MUFA. In T3, a positive correlation (*p* < 0.01) was observed for all FAs with the exception of C18:0 for which a moderate negative correlation (*p* <0.01, r= −0.387)) was observed. In T3, the Pearson’s correlation coefficient was low-moderate (r = 0.1–0.5) with the exception of C12:0 (r = 0.635), C16:0 (r = 0.724), C20:5n-3 (r = 0.763) and Σtotal SFA (r = 0.605) for which a strong correlation was observed. At both sampling times T1 and T3, the FA with the highest strong positive correlation (T1, r = 0.859 and T3, r = 0.763) was C20:5n-3.

The corresponding percentiles of FAs during T1 and T3 were calculated and showed in Table 2 and Table 3 and expressed as either an absolute concentration or a percentage of total FAs. Consequently, our reference ranges for relative concentration can be compared to the results expressed as mean ± SD from other European studies [14,15,19,38,39] shown in Table 4. The levels of individual FAs that were distinct from the 2.5 or 97.5 percentiles of the present study are indicated in Table 4.

### 3.3. Influence of Maternal Factors on Fatty Acid Serum Profiles

Multiple linear regression models of the influence of maternal determinants on total FAs, total SFA, total MUFA, total n-6 PUFA, total n-3 PUFA, LA, DHA, and AA are shown in Table 5. It can be observed that women with BMI > 30 kg/m^2^ had significantly higher levels of total SFA, total MUFA and AA in both trimesters of pregnancy. Moreover, educational level and ethnicity were significantly associated with higher values of total n-3 PUFA and DHA in T1, and lower values of total SFA, total MUFA and AA in T3. In T1, only low physical activity was associated with lower values of DHA. However, DHA and total n-3 PUFA were significantly higher in T3 at better diet quality and older age.

## 4. Discussion

The results of present study provide a reference range for the vast majority of serum circulating FAs and FAs ratios in 479 healthy pregnant women from the ECLIPSES study [28], assessed both in T1 and T3 of gestation. These results complement the few studies on reference ranges of FAs that exist in pregnant women for different moments of gestation, and add a new measurement with FAs index. Furthermore, this study describes the variation of FA levels in serum according to some maternal factors such as age, obesity, sedentary lifestyle, lower educational level, low diet quality, parity and non-Spanish ethnicity.

In general, any laboratory test is compared with reference ranges in order to assess the degree of compliance with normality levels, both individually in the clinical follow-up of the subject and collectively in epidemiological studies [9]. Adequate levels of FAs in T1 are of great importance considering the part they play in the onset of neurogenesis [7,8]. EPA and DHA are particularly critical for supporting the development of the central nervous system, retinal photoreceptors and immune system [6]. Nevertheless, few investigations have reported reference intervals for serum individualized FA concentrations, mainly due to the technical complexity of FAs evaluation compared to other nutrients or biomarkers. In the scarce literature available, FA reference ranges for serum are reported in non-pregnant adults [10,11,12,13]. Geographical region, sex, age, physio-pathological conditions [13,22] such as pregnancy [17] and dietary pattern have been seen to lead to some discrepancies. Kish-Trier et al. [40] and Mayo Clinic [12] separately provided reference intervals in age groups from the North American population with somewhat different results. Abdelmagid et al. [11] also reported significant differences between Canadian male and female adults in such FAs as LA or DHA. For pregnant women, reference values have only been described in a Brazilian cohort of 225 healthy pregnant women [16] and in a Norwegian cohort of 247 healthy pregnant women [17]. However, the concentration units (relative terms) and type of sample (erythrocyte membrane) evaluated in these two cohorts cannot be compared with our work methodology and our results. In this regard, reference intervals should be developed using a more uniform and systematic process. In any case, our study provides new information on the European Mediterranean region with socioeconomic, cultural and dietary characteristics different from the previous ones.

Depending on concentration units, the reference intervals of individual FAs in serum can be expressed as either absolute concentrations or percentages of total FA. To the best of our knowledge, no study has reported serum reference ranges in both types of concentration units in healthy pregnant women. To make our results comparable with those of previous studies, reference ranges were reported as relative concentrations because very little literature reports absolute values in pregnant women. Correlations between FA concentrations expressed in absolute and relative values were low-moderate (r = 0.1–0.5) with the exception of C12:0, C16:0, Total SFA and C20:5n-3 for which a strong correlation was observed (r > 0.5). Identically to our results, other studies have already noted poor correlations between absolute and relative concentrations [40,41,42,43] in non-pregnant adults. It is postulated that differences between percentages and concentrations depended on individual FA-characteristics [43]. Moreover, the difference in the correlation could be due to the fact that the percentage of individual FA is calculated based on the whole set of FAs detected, however absolute concentration of individual FA is not dependent upon the relative abundance of other FAs. For this reason, we believe that the measurement expressed in absolute terms would facilitate comparisons among studies.

Our relative results were compared with those reported in other European studies (Table 3). As expected, in the Mediterranean diet pattern Σtotal MUFA levels appear to be higher in the study from Spain [19] and lower in non-Spanish countries such as the study from the Netherlands [14,38]. Surprisingly, similar levels of Σtotal MUFA have also been observed in Germany [15] and in UK [39], which are not characterized by following a Mediterranean pattern diet. DHGLA and AA values were higher in the Netherlands than other countries. Normally, the production of inflammatory mediators such as AA is associated with the course of pregnancy. However, abnormally high levels have been related to pathological pregnancy complications such as preeclampsia, premature labor, fetal growth, among others (especially in women with risk factors for overweight and obesity) [27]. Some of these complications occur during T3, however Otto et al. [14] measured FA during T1. Furthermore, certain maternal factors such as obesity predispose to suffer a greater number of complications and the study of Vlaardingerbroek. et al. [38] did not report the BMI of their pregnant women population. In addition to the above, DHA concentrations from the Netherlands are higher than those reported in the other studies. Possible explanations for increased DHA in the Netherlands might be the high intake of n-3 supplements or the higher consumption of seafood per capita [17]. Finally, the differences in the measurement time window, sample size and FA assay protocol could explain the difference found with these authors [14,15,19,38,39]. Nevertheless, it is important to note that absolute concentrations are more useful and facilitate comparisons between studies and the recommendation for future studies is to report FA levels as absolute units. Further, absolute values consist in an individual measure that assure an economic analysis compared with relative values that require the measurement of all circulating FAs in serum. In this regard, absolute analysis could be included in routine clinical practice of laboratories and not be limited to research studies.

Regarding the evolution of FA levels between T1 and T3, we conclude in an article from the ECLIPSES study, that serum FA profile changed during the gestational period: Σtotal SFA, Σtotal MUFA and Σtotal n-6 PUFA increased during pregnancy, whereas essential FAs such AA and EPA decrease and DHA remains unchanged [44]. It is well-known that the composition in serum of essential FAs such n-6 PUFA and n-3 PUFA is closely linked to dietary FA consumption [18,19]. However, Volk et al. [45] showed that dietary and plasma SFA and MUFA are not related. In addition, some authors reported likewise to our results that FAs intake and the dietary pattern remained similar throughout pregnancy [14,29]. In this sense, diet would not explain the variations observed in serum FA composition and levels, including the lack of correlation between absolute and relative concentrations in our results, which could be related to other factors in combination with physiological changes in plasma volume during pregnancy.

We found that the women with obesity, sedentary lifestyle, lower educational level and those of non-Spanish ethnicity had higher levels of total SFA, total MUFA and AA, and lower levels of total n-3 PUFA and DHA. Factors such as obesity and sedentary lifestyle, could favor the presence of proinflammatory FAs such as AA. Ma et al. showed that overweight, smoking and alcohol drinking can increased the endogenous synthesis of SFA [46]. Other studies showed that variations in FA concentrations could be affected by the genetically conditions, such as genotype expression of fatty acid desaturase [25,47]. Overall, it is important to note that lifestyle, sociodemographic and genetic factors can affect normal FA reference intervals in pregnancy. Therefore, serum FA measure could be considered as an integration of dietary intake and individual biological response [46]. Consequently, absolute percentiles of FAs for 95% of the population could assist the evaluation of FA status and the identification of women at risk of either under exposure (≤2.5% percentile) or over exposure (≥97.5% percentile) to an individual FA.

One of the strengths of this study is that it is the first one to define individual SFA, MUFA, and PUFA reference ranges in absolute and relative units in a large sample of healthy women at the beginning and end of pregnancy. The representativeness of the sample size emphasizes the validity of the proposed reference values in the present study. All data were collected by the same trained researchers, and all FA analyses were performed in the same research laboratory. Furthermore, FAs were analyzed by GC-MS which is the more recently clinically validated method for individual FA quantitation [40] and can be applied for fully automated FA profiling in serum samples during dietary studies [35]. Further, our results would facilitate future research into the role of high/low concentrations of individual FAs in obstetric and fetal pathologies. Nevertheless, it does have several limitations. First, FAs were not analyzed in the second trimester of pregnancy and it would have been interesting to monitor the whole gestational period. Second, we were unable to assess all FAs in our sample. One of these was ALA, one of the main n-3 PUFAs, which may explain why the n-3 PUFA relative levels in our study were lower than those in other countries. Third, it is difficult to establish a comparison between countries because the FA profile analyzed in total serum/plasma lipids can vary from different populations, included in the same country; further the measurement of FAs in all circulating lipids differ from FA profile performed in circulating triglycerides, phospholipids or esters of cholesterol.

## 5. Conclusions

The present study provides the serum reference interval for FA levels in a large sample of pregnant women from a Mediterranean region. The data are reported in the first and third trimesters of pregnancy, expressed both as absolute values of their serum concentrations, and as relative values in terms of the percentage of total FA. The percentiles of reference ranges proposed will be a useful tool for assessing the degree of adequacy of FAs in pregnant women, in both individual monitoring of pregnancy, and population-based epidemiological studies. However, every region must provide their own reference ranges in accordance with the characteristics of their population. Further research is needed to identify the personal and environmental factors that contribute to a healthy diet during pregnancy.

## Figures and Tables

**Table 1:** Sociodemographic and lifestyle characteristics of pregnant women (*n* = 455).

General Characteristics	Summary Statistics
Maternal age (years) ^a^	30.6 ± 5.01
Country of origin, Spain (%)	84.1
Primipara (%)	37.5
Gestational age (weeks) ^a^	39.8 ± 1.08
BMI (kg/m^2^) at first trimester (%)	
18.5–24.9 (normal weight)	62.2 (22.10 ± 1.76) ^a^
25.0–29.9 (overweight)	25.3 (27.32 ± 1.33) ^a^
≥30 (obesity)	12.5 (33.31 ± 2.91) ^a^
Gestational weight gain (kg) ^a^	10.86 ± 3.66
Maternal educational level (%)	
Low (primary or less)	30.1
Medium (high school)	38.3
High (university or more)	31.6
Occupation (%)	
Student	2.4
Employed	87.1
Unemployed	10.5
Smoking status (%)	
Smoker	15.3
Non-Smoker	69.5
Ex-Smoker	15.3
Maternal alcohol consumption (%)	14
Physical Activity (METs/week) (%)	
Low (<600)	56.4
Moderate (≥600–2999)	39.4
High (≥3000)	4.2
SQDI (score) ^a^	9.73 ± 2.64

^a^ Mean ± standard deviation. Abbreviation: BMI, body mass index; SQDI, Spanish Diet Quality Index; METs, Metabolic equivalents.

**Table 2:** Absolute concentration and percentile (µmol/L) distribution of fatty acid in maternal serum during the first (T1) and third (T3) trimesters of pregnancy.

Fatty Acids		Absolute Concentration (µmol/L) *	Absolute Percentiles (µmol/L)								
			P_2.5_	P_5_	P_10_	P_25_	P_50_	P_75_	P_90_	P_95_	P_97.5_
SFA											
Lauric acid (C12:0)	T1, *n* = 469	40.14 ± 10.50	29.22	30.18	31.67	34.04	37.30	42.45	51.76	60.12	71.80
	T3, *n* = 466	60.19 ± 28.26	32.52	34.51	36.73	40.91	51.10	69.71	97.49	121.81	146.67
Myristic acid (C14:0)	T1, *n* = 471	118.16 ± 49.07	57.95	63.99	69.33	83.20	107.42	139.15	188.35	228.58	260.84
	T3, *n* = 470	207.80 ± 80.31	93.68	107.69	117.21	149.71	194.20	249.20	321.04	367.13	413.69
Palmitic acid (C16:0)	T1, *n* = 467	2904.40 ± 1403.47	1339.95	1463.74	1627.03	1937.08	2582.35	3500.79	4704.88	5573.20	7610.80
	T3, *n* = 466	8511.32 ± 4293.76	2477.62	3017.91	3880.25	5507.61	7500.15	10,738.06	14,966.15	17,376.53	18,980.26
Stearic acid (C18:0)	T1, *n* = 470	690.31 ± 208.05	398.5	435.26	465.17	547.46	639.97	804.96	1019.98	1139.45	1211.48
	T3, *n* = 472	808.08 ± 202.10	474.67	522.63	569.76	664.25	779.99	930.30	1081.33	1174.58	1302.64
Σ Total SFA	T1, *n* = 467	3765.31 ± 1614.17	1884.32	2073.77	2267.31	2646.97	3408.43	4504.30	5907.35	6961.68	8802.81
	T3, *n* = 467	9625.65 ± 4574.33	3171.60	3756.01	4644.10	6401.81	8574.46	11,903.47	16,488.0	18,902.97	20,791.60
MUFA											
Palmitoleic acid (C16:1n-7)	T1, *n* = 465	186.37 ± 45.88	119.70	125.68	136.35	151.80	180.84	211.59	250.73	283.54	296.39
	T3, *n* = 468	256.66 ± 89.61	100.78	134.70	163.33	194.08	245.46	303.10	378.4	422.99	487.91
Oleic acid (C18:1n-9)	T1, *n* = 466	1444.74 ± 461.90	817.01	881.34	959.76	1097.08	1381.89	1666.88	2044.13	2333.50	2694.42
	T3, *n* = 466	2843.85 ± 1251.92	1083.25	1319.01	1507.87	1915.78	2602.09	3514.33	4400.45	5507.76	6221.64
Σ Total MUFA	T1, *n* = 466	1634.54 ± 500.21	959.91	1020.19	1104.29	1257.50	1555.50	1900.07	2275.35	2596.12	2979.46
	T3, *n* = 467	3116.39 ± 1330.86	1254.75	1475.81	1711.59	2157.40	2860.01	3823.08	4920.80	6034.19	6668.84
n-6 PUFA											
LA (C18:2n-6)	T1, *n* = 466	3355.67 ± 1230.19	1602.64	1756.72	1927.14	2439.91	3213.76	4097.77	5012.97	5710.44	6211.58
	T3, *n* = 470	6321.14 ± 2786.02	2408.12	2737.37	3282.10	4194.24	5722.28	7957.48	10,332.80	11,602.18	13,327.69
DHGLA (C20:3n-6)	T1, *n* = 469	229.99 ± 90.64	101.13	114.98	127.81	165.93	216.25	280.67	360.30	407.91	450.08
	T3, *n* = 471	246.20 ± 85.27	116.31	129.41	146.81	184.03	233.43	300.70	363.36	397.26	445.50
AA (C20:4n-6)	T1, *n* = 473	830.82 ± 276.15	421.52	455.60	520.47	631.14	791.72	991.31	1247.92	1398.0	1523.56
	T3, *n* = 469	722.63 ± 220.05	387.52	423.29	472.62	560.47	692.0	855.29	1035.63	1114.09	1223.84
Σ Total n-6 PUFA	T1, *n* = 465	4433.77 ± 1469.64	2325.77	2420.11	2697.52	3325.21	4219.65	5333.68	6482.27	7114.28	7735.74
	T3, *n* = 469	7278.06 ± 2919.04	3126.65	3435.44	4072.94	5029.21	6758.36	9100.77	11,346.52	12,917.44	14,348.43
n-3 PUFA											
EPA (C20:5n-3)	T1, *n* = 467	35.03 ± 23.95	7.80	9.51	11.58	17.67	29.30	45.36	74.50	87.17	103.34
	T3, *n* = 470	23.88 ± 16.93	3.96	5.07	6.35	11.10	19.04	32.31	47.78	59.45	68.46
DHA (C22:6n-3)	T1, *n* = 473	240.28 ± 73.47	119.02	134.43	151.61	185.65	234.05	291.00	340.30	381.36	406.58
	T3, *n* = 474	236.60 ± 71.77	123.02	132.54	150.80	182.27	224.54	283.39	336.38	374.51	396.16
Σ Total n-3 PUFA	T1, *n* = 473	276.98 ± 94.07	129.01	149.88	169.46	205.28	264.05	332.67	414.77	469.19	495.58
	T3, *n* = 473	260.52 ± 84.33	133.12	141.08	160.92	194.55	250.85	315.61	377.51	426.69	454.23
Total FAs	T1, *n* = 464	10,073.15 ± 3349.07	5589.89	5808.29	6420.65	7561.30	9551.76	11,982.98	14,557.61	16,156.53	18,491.35
	T3, *n* = 468	20,480.82 ± 8335.23	8214.61	9780.29	10,719.47	14,577.32	18,797.72	25,341.41	31,644.12	36,078.82	41,359.35
n-6/n-3 ratio ^(a)^	T1, *n* = 471	16.80 ± 5.19	8.39	9.25	10.54	13.23	16.39	19.70	24.25	27.01	29.61
	T3, *n* = 471	29.29 ± 11.52	11.83	13.44	15.77	20.98	27.69	36.54	46.83	51.60	54.88
AA/EPA ratio	T1, *n* = 469	31.77 ± 19.08	6.31	7.66	11.10	17.73	27.44	42.68	57.56	72.19	80.34
	T3, *n* = 470	44.09 ± 30.61	9.19	10.80	13.36	22.56	37.83	57.30	92.27	113.43	133.61
AA/(EPA + DHA)	T1, *n* = 474	3.12 ± 0.87	1.65	1.83	2.05	2.49	3.05	3.73	4.32	4.53	5.06
	T3, *n* = 475	2.96 ± 0.91	1.46	1.61	1.82	2.30	2.85	3.56	4.14	4.55	4.99
LA/DHGLA ratio	T1, *n* = 469	15.45 ± 5.12	7.40	8.03	9.67	11.84	14.80	18.55	23.20	25.04	27.86
	T3, *n* = 471	26.59 ± 10.58	11.59	12.97	14.44	18.71	24.20	32.78	42.46	47.05	51.60
EFA index	T1, *n* = 473	2.91 ± 0.64	1.77	1.94	2.11	2.49	2.85	3.30	3.74	4.05	4.37
	T3, *n* = 470	2.53 ± 0.90	1.20	1.30	1.48	1.90	2.39	3.07	3.81	4.29	4.71

P: Percentile; T1: First trimester of pregnancy; T3: Third trimester of pregnancy. LA, Linoleic acid; DHGLA, Dihomo-γ-linolenic acid; AA, Arachidonic acid; EPA, Eicosapentaenoic acid; DHA, Docosahexaenoic acid. SFA, saturated fatty acids; MUFA, monounsaturated fatty acids; n-6 PUFA, omega-6 polyunsaturated fatty acid; n-3 PUFA, omega-3 polyunsaturated fatty acid; total FAs, total fatty acids = Σ total SFA + Σ total MUFA + Σ total n-6 PUFA + Σ total n-3 PUFA; (^a^) n-6/n-3 ratio = Σtotal n-6 PUFA/Σtotal n-3 PUFA; EFA index, Essential fatty acid index. (*): Fatty acid absolute concentration is presented as mean ± standard deviation (SD).

**Table 3:** Relative concentration and percentile (%) distribution of fatty acid in maternal serum during the first (T1) and third (T3) trimester of pregnancy.

Fatty Acids		Relative Concentration (% of Total FAs) *	Relative Percentiles (%)								
			P_2.5_	P_5_	P_10_	P_25_	P_50_	P_75_	P_90_	P_95_	P_97.5_
SFA											
Lauric acid (C12:0)	T1, *n* = 472	0.42 ± 0.13	0.20	0.23	0.27	0.33	0.40	0.49	0.60	0.65	0.74
	T3, *n* = 470	0.34 ± 0.20	0.12	0.14	0.16	0.20	0.28	0.40	0.62	0.79	0.94
Myristic acid (C14:0)	T1, *n* = 472	1.17 ± 0.32	0.65	0.71	0.80	0.94	1.12	1.37	1.62	1.74	1.90
	T3, *n* = 473	1.07 ± 0.36	0.49	0.56	0.67	0.81	1.02	1.29	1.59	1.76	1.97
Palmitic acid (C16:0)	T1, *n* = 471	28.09 ± 4.52	20.29	21.40	22.60	24.83	27.75	30.87	33.72	36.61	38.10
	T3, *n* = 475	40.89 ± 6.82	28.06	30.81	32.88	36.17	40.47	44.98	49.74	52.76	57.08
Stearic acid (C18:0)	T1, *n* = 475	6.88 ± 1.06	4.41	4.41	4.90	5.42	6.23	7.01	7.64	8.14	8.47
	T3, *n* = 474	4.20 ± 1.04	2.49	2.65	2.91	3.48	4.09	4.81	5.57	6.27	6.76
Σ Total SFA	T1, *n* = 470	36.57 ± 4.38	28.84	30.01	30.59	33.49	36.55	39.34	42.02	44.15	45.65
	T3, *n* = 474	46.51 ± 6.27	35.89	37.41	39.08	42.10	46.06	50.42	54.30	58.07	62.08
MUFA											
Palmitoleic acid (C16:1n-7)	T1, *n* = 475	1.92 ± 0.45	1.14	1.25	1.39	1.62	1.87	2.19	2.58	2.78	2.96
	T3, *n* = 472	1.35 ± 0.45	0.61	0.72	0.81	1.02	1.33	1.60	1.91	2.23	2.52
Oleic acid (C18:1n-9)	T1, *n* = 474	14.52 ± 2.27	10.22	11.18	11.89	13.02	14.24	16.10	17.68	18.57	19.50
	T3, *n* = 471	14.16 ± 3.36	8.52	9.11	10.13	11.68	13.72	16.37	18.77	20.32	21.40
Σ Total MUFA	T1, *n* = 475	16.46 ± 2.53	11.47	12.57	13.55	14.79	16.18	18.16	19.83	20.91	21.68
	T3, *n* = 473	15.58 ± 3.51	9.50	10.49	11.32	12.93	15.14	17.84	20.19	31.81	22.98
n-6 PUFA											
LA (C18:2n-6), *n* = 446	T1, *n* = 476	33.30 ± 5.19	23.21	24.90	26.58	29.82	33.38	36.81	39.95	42.13	44.14
	T3, *n* = 476	31.13 ± 6.95	17.65	19.97	22.21	26.28	31.03	35.61	40.23	42.68	45.69
DHGLA (C20:3n-6), *n* = 450	T1, *n* = 473	2.26 ± 0.58	1.20	1.38	1.54	1.84	2.21	2.61	2.96	3.33	3.52
	T3, *n* = 474	1.27 ± 0.40	0.63	0.71	0.81	0.98	1.21	1.54	1.78	2.05	2.20
AA (C20:4n-6), *n* = 451	T1, *n* = 475	8.31 ± 2.05	4.33	4.88	5.62	7.09	8.29	9.66	10.95	11.89	12.69
	T3, *n* = 471	3.83 ± 1.33	1.79	2.03	2.27	2.91	3.62	4.54	5.69	6.30	7.55
Σ Total n-6 PUFA, *n* = 444	T1, *n* = 474	44.35 ± 5.07	33.62	36.25	38.20	41.05	44.59	47.44	50.80	52.94	54.46
	T3, *n* = 476	36.50 ± 7.27	21.78	24.07	27.36	31.43	36.47	40.95	45.85	48.62	50.86
n-3 PUFA											
EPA (C20:5n-3), *n* = 446	T1, *n* = 467	0.36 ± 0.24	0.07	0.11	0.13	0.18	0.30	0.46	0.66	0.86	1.04
	T3, *n* = 470	0.13 ± 0.10	0.02	0.03	0.04	0.06	0.10	0.16	0.27	0.33	0.39
DHA (C22:6n-3), *n* = 456	T1, *n* = 474	2.24 ± 0.66	1.25	1.49	1.63	1.97	2.37	2.85	3.35	3.58	3.87
	T3, *n* = 472	1.24 ± 0.46	0.58	0.65	0.73	0.92	1.17	1.50	1.89	2.08	2.41
Σ Total n-3 PUFA	T1, *n* = 470	2.77 ± 0.83	1.41	1.62	1.79	2.16	2.67	3.28	3.93	4.35	4.74
	T3, *n* = 470	1.36 ± 0.53	0.64	0.70	0.78	0.98	1.27	1.66	2.12	2.42	2.67
n-6/n-3 ratio ^(a)^	T1, *n* = 472	16.93 ± 5.27	8.39	9.25	10.54	13.23	16.40	19.70	24.41	27.53	29.67
	T3, *n* = 472	29.54 ± 11.66	11.83	13.45	15.78	21.02	27.71	36.63	46.99	51.68	55.53
AA/EPA ratio	T1, *n* = 469	31.68 ± 19.01	6.31	7.66	11.10	17.73	27.44	42.68	57.56	72.19	80.34
	T3, *n* = 470	45.25 ± 32.05	9.19	10.80	13.36	22.56	37.83	57.30	92.27	113.43	133.61
AA/(EPA + DHA)	T1, *n* = 474	3.12 ± 0.87	1.65	1.83	2.05	2.49	3.05	3.73	4.32	4.53	5.06
	T3, *n* = 475	2.96 ± 0.91	1.46	1.61	1.82	2.30	2.85	3.56	4.14	4.55	4.99
LA/DHGLA ratio	T1, *n* = 471	15.69 ± 5.37	7.41	8.03	9.70	11.84	14.86	18.57	23.27	25.22	28.98
	T3, *n* = 472	26.64 ± 10.69	11.59	12.97	14.46	18.72	24.22	32.87	42.61	47.25	51.65
EFA index	T1, *n* = 473	2.93 ± 0.64	1.78	1.95	2.13	2.51	2.87	3.32	3.77	4.07	4.40
	T3, *n* = 470	2.55 ± 0.90	1.20	1.31	1.48	1.91	2.40	3.08	3.83	4.30	4.74

P: Percentile; T1: First trimester of pregnancy; T3: Third trimester of pregnancy. LA, Linoleic acid; DHGLA, Dihomo-γ-linolenic acid; AA, Arachidonic acid; EPA, Eicosapentaenoic acid; DHA, Docosahexaenoic acid. SFA, saturated fatty acids; MUFA, monounsaturated fatty acids; n-6 PUFA, omega-6 polyunsaturated fatty acid; n-3 PUFA, omega-3 polyunsaturated fatty acid; (^a^) n-6/n-3 ratio = Σtotal n-6 PUFA/Σtotal n-3 PUFA; EFA index, Essential fatty acid index. (*): Fatty acid relative concentration is presented as mean ± standard deviation (SD).

**Table 4:** A comparison of reported concentrations (%) of total fatty acids in maternal serum from different European countries.

Fatty Acids		Our Reference Intervals (T1, *n* = 472; T3, *n* = 468)	Montes et al. [19] (*n* = 170) (Spain)	Otto et al. [14] (*n* = 23) (The Netherlands)	Enke et al. [15] (*n* = 55) (Germany)	Vlaardingerbroek et al. [38] (*n* = 172) (The Netherlands)	Wheeler et al. [39] (*n* = 142) (United Kingdom)
		Percentiles (2.5–97.5%)	Mean ± SD				
SFA							
Myristic acid (C14:0)	T1	0.65–1.90	-	-	-	-	
	T3	0.49–1.97	-	-	1.21 ± 0.40	-	1.21 ± 0.03
Palmitic acid (C16:0)	T1	20.29–38.10	-	-	-	-	
	T3	28.06–57.08	-	-	30.20 ± 2.31 *	-	25.9 ± 0.17 *
Stearic acid (C18:0)	T1	4.41–8.47	-	-	-	-	
	T3	2.49–6.76	-	-	5.59 ± 0.78	-	5.84 ± 0.05
Σ Total SFA	T1	28.84–45.65	30.34 ± 2.06	44.14 ± 0.46	-	45.06 ± 0.09	
	T3	25.89–62.08	-	-	67.80 ± 4.77 ⱡ	45.80 ± 0.07	33.0 ± 0.20
MUFA							
Palmitoleic acid (C16:1n-7)	T1	1.14–2.96	-	-	-	-	
	T3	0.61–2.52	-	-	-	-	2.41 ± 0.07
Oleic acid (C18:1n-9)	T1	10.22–19.50	-	-	-	-	
	T3	8.52–21.40	-	-	23.90 ± 2.45 ⱡ	-	22.2 ± 0.40 ⱡ
Σ Total MUFA	T1	11.47–21.68	23.29 ± 3.62 ⱡ	12.05 ± 0.25	-	11.20 ± 0.08 *	
	T3	9.50–22.98	-	-	29.20 ± 2.93 ⱡ	11.99 ± 0.10	28.8 ± 0.25 ⱡ
n-6 PUFA							
LA (C18:2n-6)	T1	23.21–44.14	32.16 ± 4.16	20.48 ± 0.48 *	-	21.28 ± 0.18 *	
	T3	17.65–45.69	-	-	23.50 ± 3.41	21.78 ± 0.18	27.1 ±0.29
DHGLA (C20:3n-6)	T1	1.20–3.52	1.55 ± 0.56 *	3.53 ± 0.17 ⱡ	-	3.22 ± 0.05	
	T3	0.63–2.20	-	-	1.55 ± 0.32	3.46 ± 0.04 ⱡ	7.88 ± 0.20 ⱡ
AA (C20:4n-6)	T1	4.33–12.69	7.33 ± 1.74	9.34 ± 0.35	-	9.64 ± 0.11	
	T3	1.79–7.55	-	-	3.83 ± 0.89	8.15 ± 0.10 ⱡ	5.40 ± 0.08
Σ Total n-6 PUFA	T1	33.62–54.46	42.56 ± 4.22	34.88 ± 0.62	-	-	
	T3	21.78–50.86	-	-	-	-	34.7 ± 0.31
n-3 PUFA							
EPA (C20:5n-3)	T1	0.07–1.04	0.39 ± 0.30	0.62 ± 0.11	-	0.58 ± 0.02	
	T3	0.02–0.39	-	-	0.24 ± 0.10	0.34 ± 0.02	0.32 ± 0.02
DHA (C22:6n-3)	T1	1.25–3.87	2.68 ± 0.62	3.93 ± 0.22 ⱡ	-	3.88 ± 0.07 ⱡ	
	T3	0.58–2.41	-	-	1.21 ± 0.35	3.74 ± 0.06 ⱡ	2.07 ± 0.04
Σ Total n-3 PUFA	T1	1.41–4.74	3.69 ± 0.87	5.35 ± 0.33 ⱡ	-	-	
	T3	0.64–2.67	-	-	1.61 ± 0.46	-	2.72 ± 0.06 ⱡ
n-6/n-3 ratio ^(a)^	T1	8.39–29.61	12.16 ± 3.04	6.44 ± 0.29 *	-	-	
	T3	11.83–54.88	-	-	-	-	
AA/(EPA + DHA)	T1	1.65–5.06	-	-	-	-	
	T3	1.46–4.99	-	-	-	-	2.32 ± 0.04
EFA index	T1	1.78–4.40	-	3.48 ± 0.09	-	3.62 ± 0.04	
	T3	1.20–4.74	-	-	-	3.34 ± 0.04	

T1: First Trimester of pregnancy; T3: Third trimester of pregnancy. LA, Linoleic acid; DHGLA, Dihomo-γ-linolenic acid; AA, Arachidonic acid; EPA, Eicosapentaenoic acid; DHA, Docosahexaenoic acid. SFA, saturated fatty acids; MUFA, monounsaturated fatty acids; n-6 PUFA, omega-6 polyunsaturated fatty acid; n-3 PUFA, omega-3 polyunsaturated fatty acid; (^a^) n-6/n-3 ratio = Σtotal n-6 PUFA/Σtotal n-3 PUFA; EFA index, Essential fatty acid index. (-) indicates that the fatty acid is not reported. The (*) and (ⱡ) denote: under 2.5 percentile and over 97.5 percentile of the present study, respectively.

**Table 5:** Multiple linear regression of potential factors related to maternal serum fatty acids composition in the first (T1) and third (T3) trimesters of pregnancy.

Fatty Acids												
	Total FA			Total SFA			Total MUFA			Total n-6 PUFA		
Maternal factors in T1	B	SE	*p*	B	SE	*p*	B	SE	*p*	B	SE	*p*
Maternal Age	5.8	44.0	0.895	−14.5	21.1	0.494	0.3	6.6	0.959	2.1	19.6	0.915
Occupation	100.9	423.8	0.812	115.2	202.1	0.569	32.3	63.8	0.614	22.2	188.3	0.906
Educational level	−152.8	299.0	0.610	16.2	143.1	0.910	47.1	45.4	0.300	−124.4	132.8	0.350
Ethnicity	395.4	578.6	0.495	334.0	275.5	0.226	108.4	86.4	0.211	93.5	256.8	0.716
Parity	403.7	412.6	0.329	417.3	198.2	0.036 *	116.5	62.4	0.063	115.2	183.0	0.530
BMI	97.3	43.2	0.025	56.1	20.4	0.006 *	13.2	6.5	0.044 *	34.7	19.2	0.072
Gestational weight gain	49.8	48.3	0.304	−27.5	23.1	0.236	−0.1	7.3	0.992	−19.9	21.5	0.354
Smoking	−148.3	256.3	0.563	−4.1	123.0	0.973	−35.1	39.0	0.369	−99.6	113.8	0.382
Alcohol consumption	−2790.3	3316.8	0.401	−1917.9	1589.0	0.228	−567.4	502.5	0.260	−468.6	1473.4	0.751
Physical Activity	−145.6	333.0	0.662	6.4	159.4	0.968	−25.2	50.4	0.617	−118.3	147.8	0.424
Diet Quality	106.3	73.4	0.149	55.8	35.1	0.113	16.5	11.1	0.138	21.0	32.6	0.520
	R^2^ = 0.056, F_11,280_= 1.502; *p*= 0.130			R2 = 0.081, F_11,281_= 2.248; *p*= 0.012 *			R^2^ = 0.060, F_11,281_= 1.632; *p*= 0.089			R^2^ = 0.041, F_11,281_= 1.101; *p*= 0.360		
Maternal factors in T3												
Maternal Age	191.2	108.3	0.079	37.0	59.8	0.536	37.2	17.9	0.080	36.5	38.2	0.340
Occupation	−1059.6	1058.4	0.318	−878.9	576.9	0.129	−306.2	174.3	0.511	154.0	369.9	0.677
Educational level	−1453.6	737.6	0.050	−1002.6	406.6	0.014 *	−81.1	123.4	0.026 *	−446.0	259.0	0.086
Ethnicity	−1851.1	1429.2	0.196	−922.6	785.1	0.241	−527.1	235.1	0.384	−761.8	500.1	0.129
Parity	−745.5	1030.0	0.470	739.1	569.5	0.195	−148.1	170.0	0.014 *	−483.3	360.8	0.181
BMI	254.1	108.4	0.020	164.3	59.5	0.006 *	44.0	17.8	0.635	57.3	37.8	0.130
Gestational weight gain	149.2	119.7	0.214	107.2	66.2	0.106	9.5	20.0	0.229	17.3	42.2	0.683
Smoking	−271.5	638.2	0.671	−386.9	354.1	0.275	−127.9	106.1	0.659	−36.2	223.8	0.872
Alcohol consumption	−4228.6	8281.3	0.610	−1697.3	4543.5	0.709	−609.0	1378.4	0.475	−2083.2	2909.4	0.475
Physical Activity	−482.1	818.8	0.557	−688.4	456.4	0.133	−97.2	136.0	0.507	−49.0	287.8	0.865
Diet Quality	130.1	182.9	0.477	89.0	100.2	0.375	20.2	30.3	0.080	58.8	64.5	0.363
	R^2^ = 0.061, F_11,283_= 1.673; *p*= 0.079			R^2^ = 0.091, F_11,282_= 2.573; *p*= 0.004 *			R^2^ = 0.091, F_11,285_= 2.587; *p*= 0.004 *			R^2^ = 0.041, F_11,284_= 1.098; *p*= 0.362		
	**Total n-3 PUFA**			**Linoleic Acid**			**Arachidonic Acid**			**Docosahexaenoic Acid**		
Maternal factors in T1	B	SE	*p*	B	SE	*p*	B	SE	*p*	B	SE	*p*
Maternal Age	2.9	1.3	0.027 *	5.6	16.5	0.735	−1.6	3.7	0.671	1.6	1.0	0.101
Occupation	−9.1	12.5	0.465	−52.1	159.1	0.744	26.7	36.1	0.460	−9.7	9.7	0.316
Educational level	23.3	8.8	0.009 *	−161.1	111.8	0.151	33.4	25.6	0.193	17.2	6.8	0.012 *
Ethnicity	42.3	16.8	0.012 *	11.5	216.7	0.958	6.8	48.6	0.889	29.1	13.0	0.026 *
Parity	13.8	12.1	0.257	81.4	154.6	0.599	70.2	35.1	0.046 *	11.2	9.3	0.233
BMI	0.7	1.3	0.600	29.0	16.2	0.074	8.6	3.6	0.018 *	0.8	1.0	0.404
Gestational weight gain	−0.2	1.4	0.880	−16.0	18.1	0.379	0.3	4.1	0.950	−0.2	1.1	0.834
Smoking	−2.6	7.6	0.736	−122.6	96.1	0.203	8.2	22.0	0.709	−3.3	5.8	0.570
Alcohol consumption	−67.8	97.8	0.489	−156.9	1245.0	0.900	−303.4	284.6	0.287	−25.1	75.6	0.740
Physical Activity	−17.9	9.8	0.068	−30.2	124.9	0.809	−64.7	28.4	0.024	−17.4	7.6	0.023 *
Diet Quality	1.0	2.2	0.645	28.8	27.5	0.296	−5.0	6.3	0.429	0.9	1.7	0.581
	R^2^ = 0.111, F_11,283_= 3.198; *p*= <0.001 *			R^2^ = 0.045, F_11,282_= 1.214; *p*= 0.277			R^2^ = 0.061, F_11,285_= 1.675; *p*= 0.078			R^2^ = 0.108, F_11,284_= 3.132; *p*= 0.001 *		
Maternal factors in T3												
Maternal Age	3.2	1.1	0.004 *	27.5	35.3	0.437	1.8	2.9	0.543	2.8	0.9	0.002
Occupation	−3.4	10.8	0.753	204.9	342.5	0.550	24.4	28.3	0.388	−0.2	9.1	0.981
Educational level	8.8	7.6	0.246	−419.4	239.4	0.081	−11.1	19.9	0.578	6.4	6.4	0.314
Ethnicity	1.6	14.5	0.915	−624.9	462.4	0.178	−96.8	38.4	0.015 *	5.2	12.2	0.674
Parity	−7.0	10.4	0.501	−406.5	334.1	0.225	1.8	27.4	0.947	−9.1	8.8	0.304
BMI	−0.2	1.1	0.855	49.3	34.9	0.160	6.0	2.9	0.036 *	−0.3	0.9	0.776
Gestational weight gain	−0.2	1.2	0.876	11.3	39.0	0.773	3.1	3.6	0.384	−0.2	1.0	0.825
Smoking	4.3	6.5	0.506	−26.4	207.1	0.899	17.7	17.2	0.303	3.0	5.5	0.582
Alcohol consumption	−86.9	84.8	0.306	−1756.6	2690.1	0.514	−161.6	221.9	0.467	−62.4	71.7	0.385
Physical Activity	−10.4	8.4	0.215	4.8	266.1	0.986	−7.4	21.9	0.737	−7.2	7.1	0.307
Diet Quality	3.6	1.9	0.055	38.9	60.0	0.517	−7.5	5.0	0.133	3.1	1.6	0.050 *
	R^2^ = 0.082, F_11,286_= 2.331; *p*= 0.009 *			R^2^ = 0.037, F _11,283_= 0.996; *p*= 0.450			R^2^ = 0.065, F_11,283_= 1.792; *p*= 0.049 *			R^2^ = 0.077, F_11,287_= 2.170; *p*= 0.016 *		

B, unstandardised coefficient; SE, standard error. (*) Level of statistical significance *p* > 0.05. Variables included in multiple linear regression: maternal age (years), occupation (student, employed, unemployed), ethnicity (Spanish, non-Spanish women), parity (no, yes), initial BMI (Kg/m^2^), gestational weight gain (Kg/m^2^), maternal smoking status (no, yes), maternal alcohol consumption (no, yes), physical activity in METs (score), diet quality (score).

## Data Availability

The data presented in this study are available on request from the corresponding author. The data are not publicly available due to restrictions of privacy.

## Supplementary Materials

The following are available online at https://www.mdpi.com/article/10.3390/nu13114037/s1, Table S1: Pearson correlations between absolute (µmol/L) and relative (% of total FAs) concentration of fatty acids in serum.
