# Supplementary material for: Trimester-Specific Reference Ranges for Saturated, Monounsaturated and Polyunsaturated Fatty Acids in Serum of Pregnant Women: A Cohort Study from the ECLIPSES Group
Source: Nutrients. 2021 Nov 12;13(11):4037. doi: 10.3390/nu13114037 (PMC8620362; doi:10.3390/nu13114037)
Supplement: Supplementary file 1 [file nutrients-13-04037-s001.zip › nutrients-1443650-supplementary.pdf]

Supplementary Table S1. Pearson correlations between absolute (μmol/L) and relative (% of total FAs) concentration of fatty acids in serum.

Fatty acids		First trimester (T1) Between absolute and relative concentration			Third trimester (T3) Between absolute and relative concentration	
		r	*p-values**		*r*	*p-values**
**SFA**						
Lauric acid (C12:0)	n= 471 – 472	0.254	**< 0.001**	n= 466 – 470	0.635	**< 0.001**
Myristic acid (C14:0)	n= 471 – 472	0.454	**< 0.001**	n= 472 – 473	0.334	**< 0.001**
Palmitic acid (C16:0)	n= 468 – 471	0.676	**< 0.001**	n= 470 – 475	0.724	**< 0.001**
Stearic acid (C18:0)	n= 472 – 475	-0.41	0.374	n= 474 – 475	-0.387	**< 0.001**
Σ Total SFA	n= 468 – 470	0.541	**< 0.001**	n= 468 – 474	0.605	**< 0.001**
**MUFA**						
Palmitoleic acid (C16:1n-7)	n= 466 – 475	0.108	0.020	n= 469 – 472	0.211	**< 0.001**
Oleic acid (C18:1n-9)	n= 466 – 474	0.084	0.071	n= 469 – 471	0.349	**< 0.001**
Σ Total MUFA	n= 466 – 475	-0.007	0.880	n= 469 – 472	0.285	**< 0.001**
**n-6 PUFA**						
LA (C18:2n-6)	n= 467 – 476	0.397	**< 0.001**	n= 473 – 474	0.370	**< 0.001**
DHGLA (C20:3n-6)	n= 471 – 473	0.477	**< 0.001**	n= 474	0.163	**< 0.001**
AA (C20:4n-6)	n= 473 – 475	0.307	**< 0.001**	n= 471 – 473	0.154	**0.001**
Σ Total n-6 PUFA	n= 469 – 474	0.169	**< 0.001**	n= 473 – 476	0.206	**< 0.001**
**n-3 PUFA**						
EPA (C20:5n-3)	n= 467 – 469	0.859	**< 0.001**	n= 464	0.763	**< 0.001**
DHA (C22:6n-3)	n= 474 – 475	0.306	**< 0.001**	n= 472 – 476	0.215	**< 0.001**
Σ Total n-3 PUFA	n= 470 – 473	0.416	**< 0.001**	n= 470 – 475	0.291	**< 0.001**

LA, Linoleic acid; DHGLA, Dihomo-γ-linolenic acid; AA, Arachidonic acid; EPA, Eicosapentaenoic acid; DHA, Docosahexaenoic acid. SFA, saturated fatty acids; MUFA, monounsaturated fatty acids; n-6 PUFA, omega-6 polyunsaturated fatty acid; n-3 PUFA, omega-3 polyunsaturated fatty acid. r, Pearson's correlation coefficient; (*) p-values < 0.01 were considered significant.
